# Preventive Effects of *Pyungwi-san* against Dextran Sulfate Sodium- and *Clostridium difficile*-Induced Inflammatory Bowel Disease in Mice

**DOI:** 10.3390/ijms20246346

**Published:** 2019-12-16

**Authors:** Meng Yang, Shambhunath Bose, Soo-Kyoung Lim, Hojun Kim

**Affiliations:** 1 Department of Rehabilitation Medicine of Korean Medicine, Dongguk University, 814 Siksa-dong, Ilsandong-gu, Goyang-si 10326, Korea; yimuziyan@naver.com (M.Y.); sklim1972@naver.com (S.-K.L.); 2 Scientific Consultant Board, BexPharm Korea Healthcare Ltd. 51, Seongsui-ro, Seongdong-gu, Seoul 04781, Korea; shambose@yahoo.com

**Keywords:** inflammatory bowel disease, colitis, gut microbiota, dextran sulfate sodium, *Clostridium difficile*, Pyungwi-san

## Abstract

Several lines of evidence indicate that inflammatory bowel disease (IBD) is associated with *Clostridium difficile* (CD) infection as a consequence of gut dysbiosis. Currently available treatments of IBD are either not very effective or have adverse effects. *Pyungwi-san* (PWS), a traditional Chinese herbal formulation, has long been used to treat gastrointestinal disorders. The present study was conducted to investigate the efficacy of PWS against dextran sulfate sodium (DSS) + CD-induced IBD in mice. The animals received DSS in drinking water for seven days to produce DSS-induced acute colitis. In the DSS + CD group, the DSS-fed animals were orally administered with CD spores twice during the DSS treatment period. We observed that exposure of DSS + CD-treated animals to PWS significantly decreased the disease activity index; prevented the shortening of colonic length and increases in spleen size and weight; restored colonic histological parameters by significantly increasing mucus thickness, crypt depth, and goblet cell numbers; protected the tight junction proteins; improved the profiles of pro-inflammatory and anti-inflammatory cytokines; and normalized the abundance ratio of the *Firmicutes/Bacteroidetes* in the gut. Thus, PWS exerted a number of protective effects on DSS + CD-induced colitis, which might be mediated via restoration of a balance in gut microbial communities.

## 1. Introduction

Inflammatory bowel disease (IBD), which includes ulcerative colitis and Crohn’s disease, is a chronic inflammatory intestinal condition manifested by abdominal pain, weight loss, diarrhea, rectal bleeding, and diffuse mucosal inflammation [1,2]. Accumulating evidence indicates that both the onset and development of IBD are influenced by multiple factors such as host genetic susceptibility, environmental influence, imbalance of gut symbiotic microorganisms, gut epithelial condition, and the host’s immune system [3].

A characteristic feature of patients suffering from IBD is the dysfunction and deformity of the intestinal epithelial tight junction (TJ), which is manifested by low-level expression of intestinal epithelial TJ-related proteins and/or their abnormal distribution [4,5]. This is further evidenced by the observation that intestinal epithelial barrier permeability increases as a consequence of disease progression of IBD [6]. Therefore, improving the integrity of the intestinal barrier by manipulating the TJ is considered a prime therapeutic target and strategy for IBD treatment [7].

Claudins and occludin are the two key proteins in the TJ [8]. The claudin proteins, which have four transmembrane domains, are considered to be the structural backbone of the TJ. In the intestine, claudin-1 along with claudins-3, -4, -5, and -8 tighten the TJ, and thereby decrease paracellular permeability [9]. Meanwhile, occludin, a 65 kDa tetraspan transmembrane membrane protein, contributes to TJ stabilization and optimal barrier function [10]. In both active and inactive *Clostridium difficile* (CD) periods, claudin-1 and occludin show significant decreases in their expression levels at the protein and mRNA levels in the colonic mucosa, which has been associated with damage of the intestinal epithelial barrier and a concomitant increase in permeability of the intestinal epithelium [11,12].

It has been reported that interleukin (IL)-1β, a pro-inflammatory cytokine, is likely to have a role in the pathogenesis of IBD [13]. A previous study identified multiple mechanisms through which IL-1β promotes intestinal pathology and suggested that targeting this cytokine may represent a useful therapeutic strategy in IBD [14]. In addition, it has been observed that two immunomodulatory cytokines, transforming growth factor (TGF)-β and IL-10, are significantly involved in maintaining a tolerogenic state in the adult human intestinal mucosa [15]. Furthermore, it has been revealed that these two proteins in the mucosa have crucial roles in preventing lipopolysaccharide (LPS)-driven, Interferon-γ (IFN-γ)-mediated epithelial damage in human colon explants [16].

Gut microbes are considered to be key factors in intestinal inflammation in IBD and a number of studies have suggested that dysbiosis occurs in IBD [17]. An accumulation of evidence indicates the presence of a broad microbial alteration pattern in IBD that is characterized by a decrease in biodiversity and species richness [18]. Furthermore, human studies have demonstrated that the abundance of specific bacteria taxa can change in IBD [17]. It has also been observed that IBD is associated with *Clostridium difficile*, an anaerobic gram-positive, spore-forming, toxin-producing bacillus that causes intestinal diseases ranging from mild diarrheal illness to severe colitis. Patients with IBD are at higher risk of susceptibility to *C. difficile* infection (CDI), have worse outcomes of CDI including increased rates of colectomy and death, and exhibit a higher recurrence rate [19].

Thus far, the therapeutic strategies adopted for combating IBD encompass treatment of colitis via administration of anti-inflammatory or immunosuppressive drugs, antibiotics, and surgery which aims to regulate immune cell-derived cytokine production. However, undesirable side effects such as allergies, fever, cramps, lymphoma, and diabetes limit the long-term use of these therapies. Thus, a lot of research has been undertaken to find alternative therapeutic strategies for IBD treatment, strategies that will be efficacious on the one hand and devoid of undesirable side effects on the other [20,21]. There is increasing evidence indicating the usefulness of herbal medicines in IBD treatments. For instance, a Huangqin decoction has been shown to ameliorate DSS-induced colitis by altering gut microbiota [22]. In addition, a traditional Chinese herbal medicine Iberogast has been reported to alleviate colitis [23]. Notably, a *Pyungwi-san* (PWS) decoction, a mixed herbal formulation composed of the extract of six herbs, has been used in clinics for thousands of years in China owing to its anti-oxidative and anti-inflammatory activities [24]. The description on PWS formula was first published in 1107 AD in the Prescriptions of Taiping Benevolent Dispensary (*Tai ping hui min he ji ju fang*) [25]. In traditional medicine, the term *Pyung* (calming) signifies calming down of all the unbalanced things. *Wi* (stomach) does not only represent the stomach organ; rather it defines the entire digestive system that removes obstruction and stagnation by elimination through the intestine [26]. On the basis of the outcome of the Chinese traditional and Western medicines-related research, the functions or therapeutic effects of all the herbs in the PWS formulation have been well documented, including those where the Chinese herbal medicinal terminologies were used [27,28,29,30]. In recent years, the pharmacological evaluation of PWS herbs has demonstrated that many of them have a significant beneficial impact that supports their traditional use. For instance, *Citri Pericarpiumpeel* has traditionally been used in Asia as a medicine for the improvement of asthma and adverse bronchial conditions or blood and cardiac circulation [31]. It has been demonstrated that *glycyrrhizin*, a bioactive triterpene saponin present in *Glycyrrhiza uralensis* of PWS formulatiion, causes strong attenuation of inflammatory responses induced by Toll like receptor (TLR) 3 and TLR 4 ligands [32]. This molecule has also been shown to exert a neuroprotective effect in the post-ischemic brain via anti-inflammatory action by inhibiting high-mobility group box 1 (HMGB1) phosphorylation and secretion [33]. Moreover, *magnolol*, the active compound of the *Magnoliae Cortex*, another component of PWS, has been shown to possess anti-inflammatory activities [34,35]. Additionally, *Rhizoma Atractylodis Macrocephalae* (RAM), a herb that is also a constituent of PWS, and its main compounds are known to exert various pharmaceutical impacts on experimental models, including anti-cancer [36,37], anti-coagulant [38], anti-ulcer [39], anti-oxidation [40], anti-inflammatory [41], and anti-adipogenic [42] effects.

Accumulating evidence indicates the usefulness of PWS in the treatment of common gastrointestinal disorders, such as gastritis, esophageal reflux, gastric or duodenal ulcers, and acute or chronic enteritis [43]. It has been revealed that RAM prevents virus-induced gastroenteritis via the protection of intestinal mucosal cells against injury and improvement in the absorption [44]. However, the effect of PWS on acute colitis has not yet been described in detail. This prompted us to investigate the therapeutic efficacy of PWS against colitis using a DSS and DSS plus *C. difficile*-induced mouse models of IBD.

## 2. Results

### 2.1. The Effect of PWS Treatment on Clinical and Tissue Parameters in DSS and DSS+ C. difficile-Induced Colitis

**Figure 1:**
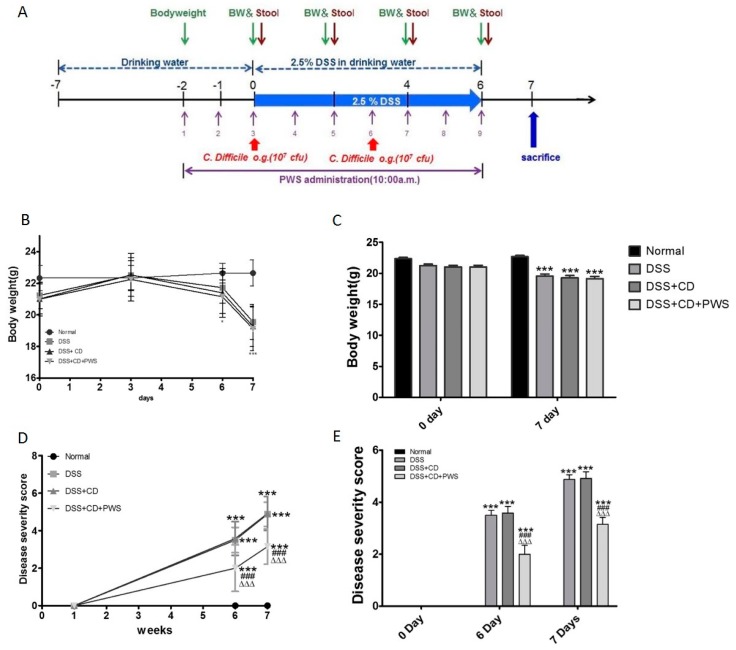
The effect of *Pyungwi-san* (PWS) treatment on clinical symptoms in mice with dextran sulfate sodium (DSS) + *Clostridium difficile* (CD)-induced colitis. Schematic representation of the experimental design. The time-frame (days) for the treatment regimen, sampling, measurements of bodyweight (bw), and animal sacrifice are depicted (**A**). Effect of PWS on the DSS + CD-induced changes in bw of mice is shown (**B**,**C**). Effect of PWS on the disease activity of DSS + CD-treated mice is demonstrated (**D**,**E**). *** p < 0.0001 versus the normal group; *^###^ p <* 0.001 versus the DSS group; *^ΔΔΔ^ p <* 0.001 versus the DSS + CD group.

No significant difference in body weight was detected among the study groups on day 0 of the experimental period (Figure 1B,C). With the exception of the normal group, an increase in body weight was observed in all experimental groups from day 0 to day 3. From day 3 onward, the bodyweight of the normal group tended to increase up to day 6 followed by stabilization on day 7. The bodyweight of all treatment groups exhibited a rapid fall from day 3 to the end of the experimental period; accordingly, body weights were found to be significantly lower in the treatment groups compared with the normal group on day 7.

As expected, no sign of disease activity was seen in the animals of the normal group throughout the experimental period (Figure 1D,E). In contrast, prominent signs of the initiation of disease activity were shown by all treatment groups on day 1, followed by rapid progression from day 1 onward. Accordingly, a very significantly high level of disease activity was observed in the animals of the DSS and DSS + CD groups compared with the normal group (*p* < 0.0001) at both days 6 and 7 of the experimental period However, treatment of the DSS + CD group with PWS significantly decreased the disease severity at both of these times (*p* < 0.001).

**Figure 2:**
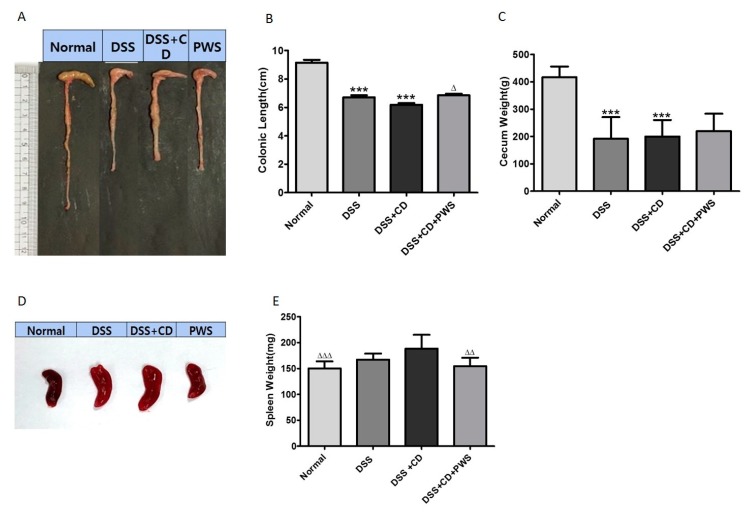
The protective role of PWS against DSS + CD-induced colitis in mice. Representative images of the measured colonic length of mice from each experimental group after sacrifice (**A**). The effect of PWS on the colonic length (**B**) and cecum weight (**C**) of DSS + CD-treated mice is shown. Representative images of the spleen of the animals from each experimental group after sacrifice (**D**). The effect of PWS on the spleen weight of DSS + CD-treated mice is demonstrated (**E**). *** p < 0.0001 versus the normal group; *^Δ^ p* < 0.05, *^ΔΔ^ p* < 0.001, and *^ΔΔΔ^* *p* < 0.001 versus the DSS + CD group.

To further assess the protective effect of PWS against DSS + CD-induced adversity, tissue parameters from samples collected from animals sacrificed animals on day 7 were analyzed. Colonic length and cecum weight were significantly lower in both the DSS and DSS + CD groups compared with those of the normal group (Figure 2A–C). However, a significant increase in colonic length was observed in the DSS + CD + PWS group, although co-treatment with PWS did not produce a significant effect on cecum weight. Treatment of animals with DSS or DSS + CD also resulted in an increase in spleen size and spleen weight, but the increases were significant only in the latter treatment (Figure 2D,E). Nevertheless, the spleen weight in the DSS + CD + PWS group was significantly lower than that in the DSS + CD group, indicating a marked response to co-treatment with PWS.

**Figure 3:**
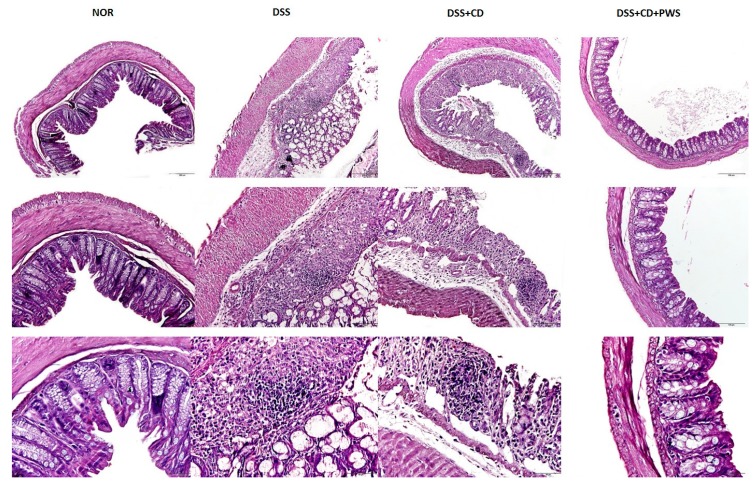
The protective effect of PWS against colonic histological damage and inflammation in DSS + CD-treated mice by hematoxylin and eosin (H&E) staining. Representative microscopic images demonstrating H&E staining of colonic tissue sections of mice from different experimental groups at a magnification of 100× (upper panel), 200× (middle panel), and 400× (lower panel).

**Figure 4:**
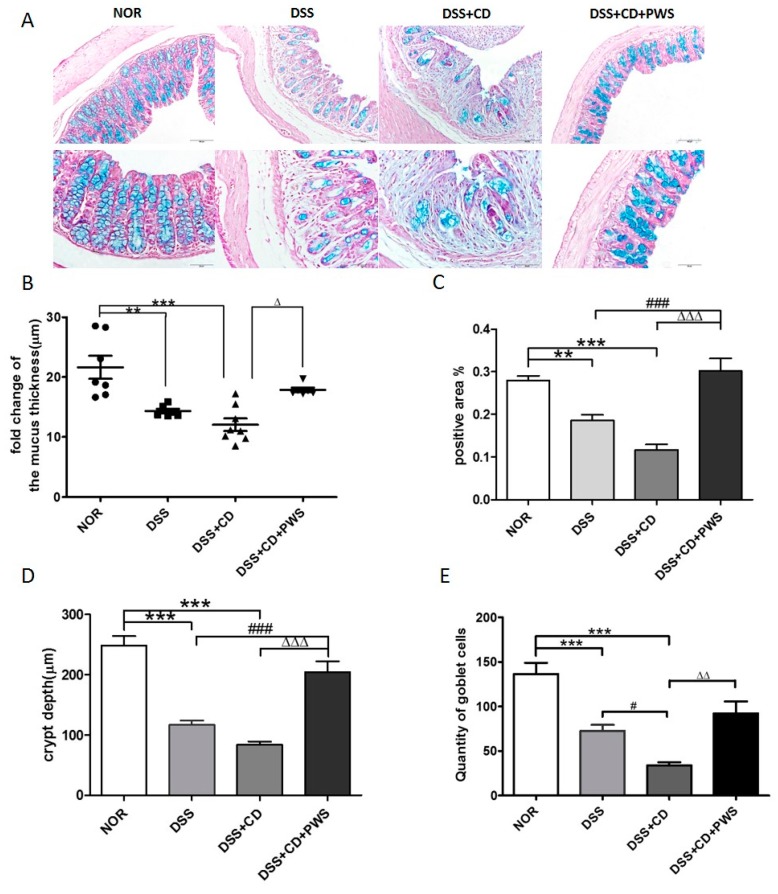
The protective effect of PWS on colon histological architecture in DSS + CD-treated mice. Representative microscopic images demonstrating Alcian blue (AB)-staining of colonic tissue sections of mice from different experimental groups at a magnification of 200× (upper panel) and 400× (lower panel). (**A**) The thickness of the inner mucus layer (**B**), proportion of AB-positive area (%) (**C**), depth of the crypt (**D**), and number of goblet cells (**E**) in colonic tissue sections were determined by the analysis of images as described in Materials and Methods. * *p* < 0.05, ** p < 0.001, and *** p < 0.0001 versus the normal group; *^#^ p* < 0.05 and *^###^ p* < 0.001 versus the DSS group; *^Δ^* *p* < 0.05, *^ΔΔ^ p* < 0.001, and *^ΔΔΔ^ p* < 0.001 versus the DSS + CD group. NOR, normal control.

To determine whether PWS treatment protects against histological damage induced by DSS or DSS + CD treatments, we performed hematoxylin and eosin (H&E) and Alcian blue (AB)-staining of the colonic tissue sections (Figure 3 and Figure 4, respectively). Our results on H&E staining showed that both the DSS and DSS + CD treatments caused inflammation, cell infiltration, crypt disruption, and ectasia with crypt abscesses, as well as an incidence of extensive epithelial hyperplasia in the colon. Interestingly, the degree of colonic damage was more in the DSS + CD group than in the DSS group. It is noteworthy that the colon lesion was less pronounced in the DSS + CD + PWS group compared with the DSS and DSS + CD groups, as noted by an observed decrease in epithelial loss and alleviation in inflammation.

Histological evaluation of the AB-stained colonic tissue was performed to investigate the structural integrity of the intestinal mucosa. As expected, exposure of mice to DSS or DSS + CD resulted in marked destruction of the colonic epithelial architecture (Figure 4A). Specifically, the thickness of the inner mucus layer of the colon was reduced markedly by both of these treatments, but more significantly reduced by DSS + CD (*p* < 0.0001) than by DSS alone (*p* < 0.001) (Figure 4B). However, a significant increase in mucus thickness was observed in the DSS + CD + PWS group. Consistent with these results, the AB-positive area that represents both neutral and acidic mucins was decreased markedly by both DSS and DSS + CD treatments, but more significantly decreased by the latter treatment (*p* < 0.0001) than the former one (*p* < 0.001) (Figure 4A,C). Nevertheless, a significant increase (*p* < 0.001) in the AB-stained area was observed in the DSS + CD + PWS group, indicating a notable response to co-treatment with PWS. In support of the above results, a significant reduction (*p* < 0.0001) in crypt depth and quantity of goblet cells was observed upon treatment with both DSS and DSS + CD (Figure 4A,D,E). However, the crypt depth was significantly enlarged (*p* < 0.001) and the goblet cell number was significantly increased (*p* < 0.01) in the DSS + CD mice co-treated with PWS.

### 2.2. Effects of PWS on Colitis-Induced Inflammatory Insult

**Figure 5:**
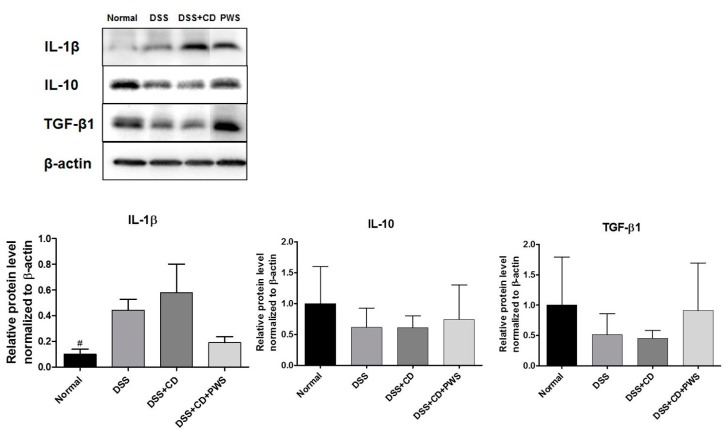
Western blot analysis showing the effect of PWS on the expression of interleukin (IL)-1β, IL-10, and transforming growth factor (TGF)-β1 in DSS + CD-treated mice. Representative blots from experiments for each protein are shown (upper panel). The density of the bands in each blot was quantified by performing densitometric analysis and was normalized to the amount of β-actin (lower panel). Data are represented as mean ± SD.

To evaluate the anti-inflammatory effects of PWS on DSS + CD-induced colitis, we focused on the pro-inflammatory cytokine IL-1β, and the anti-inflammatory cytokines IL-10 and TGF-β. The Western blot analyses of colonic tissue lysates revealed a higher expression of IL-1β and a lower expression of IL-10 and TGF-β in both the DSS and DSS + CD groups compared with those of the normal group (Figure 5). However, PWS co-treatment of DSS + CD-treated mice resulted in a decreased tissue level of IL-1β and increased tissue expression of both IL-10 and TGF-β.

### 2.3. PWS Protects Intestinal Tight Junction Proteins from Colitis-Induced Damage

**Figure 6:**
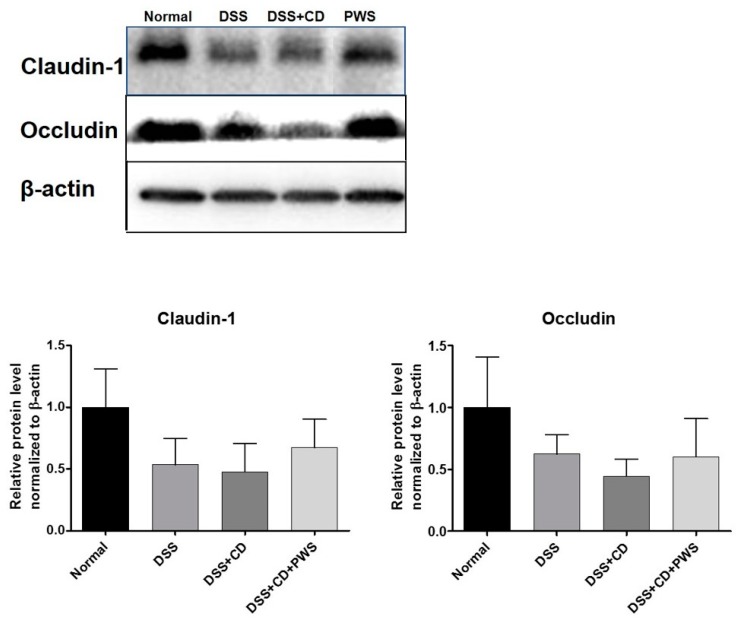
Western blot analysis showing the effect of PWS on the expression of tight junction proteins claudin-1 and occludin in DSS + CD-treated mice. Representative blots from experiments for each protein are shown (upper panel). The density of the bands in each blot was quantified by undertaking densitometric analysis and was normalized to the amount of β-actin (lower panel). Data are represented as mean ± SD.

To get an indirect measure of the extent to which the TJ is disrupted in response to DSS- and DSS + CD-induced colitis, the colonic expression of TJ markers, namely occludin and claudin-1, was analyzed. Tissue levels of claudin-1 were decreased in response to both DSS and DSS + CD treatments (Figure 6). Similarly, colonic expression of occludin was downregulated by both DSS and DSS + CD treatments, although more markedly decreased by the latter treatment compared with the former one. However, exposure of DSS + CD mice to PWS caused a marked increase in the tissue levels of both of these TJ proteins.

### 2.4. PWS Treatment Restores the Distribution of Gut Microbial Communities in Colitis

**Figure 7:**
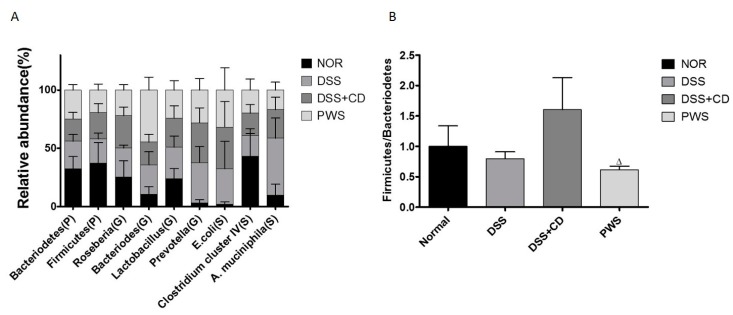
Effect of PWS on the gut microbe relative abundance in mice. Comparisons of the relative abundance of gut microbiota among the different experimental groups as indicated are shown (**A**). The compositions of the gut microbial population at phylum, genus, and species levels were compared among the treatment groups and presented as bar plots. The effects of PWS on the ratio of relative abundance between the *Firmicutes* and *Bacteroidetes* phyla among the different experimental groups are demonstrated (**B**). Data are represented as mean ± SD. *^Δ^ p* < 0.05, versus the DSS + CD group, assessed by Student’s *t* test. NOR, normal control.

The rRNA-based terminal fragment length polymorphism (T-RFLP)-based analyses of stool samples collected at the termination of the experimental period revealed a marked variation in the distribution of gut microbial population among the experimental groups (Figure 7A). At the phylum level, the population of *Bacteroidetes* was lower in the DSS + CD group than that in both the DSS and normal groups, whereas the relative abundance of *Firmicutes* was lower in both the DSS and DSS + CD groups than in the normal group, although the differences were not statistically significant. Notably, the ratio between these two phyla (*Firmicutes/Bacteroidetes*) was significantly decreased in the DSS + CD-treated mice co-treated with PWS (Figure 7B). At the genus level, the relative abundance of *Bacteroides* was insignificantly higher in the DSS group than in the normal group, but was markedly higher in the DSS + CD + PWS group than in the other groups. The relative abundance of *Prevotella* was significantly higher in both the DSS and DSS + CD groups compared with that in the normal group and was lower in the DSS + CD + PWS group than that in the DSS + CD group. At the species level, compared with the normal group, the relative abundance of *Escherichia coli* (S) was higher in both the DSS and DSS + CD groups, but the difference was significant only in the latter group. The relative abundance of *E. coli* (S) was lower in the DSS + CD + PWS group than that in both DSS and DSS + CD groups. The relative abundance of *Clostridium* cluster IV (S) was markedly lower in all the treated groups, including the DSS + CD + PWS group, compared with that in the normal group. The relative abundance of *Akkermansia muciniphila* (S) was significantly higher in the DSS group than in the normal group. Co-treatment of DSS + CD-treated mice with PWS resulted in a decrease in the abundance of *A. muciniphila* (S), although the difference was not statistically significant (*p* = 0.0695). 

## 3. Discussion

Until now, IBD has been considered an outcome of a disturbed mucosal immune response to multiple factors that lead to chronic intestinal inflammation and tissue destruction [1,45]. Notably, the current line of evidence indicates that IBD is associated with *C. difficile*, a bacterial strain that is known to be a major cause of hospital-acquired infection and is responsible for the development of symptoms ranging from diarrhea to severe colitis, sepsis, and ultimately death. Consistent with these findings, there is an accumulation of evidence indicating an increasing incidence of CDI and associated pathogenicity in IBD patients [46,47]. It has been suggested that the relationship between *C. difficile* and IBD is mediated by a series of events including recurrent hospital admissions, which are a known risk factor for acquisition of *C. difficile* and CDI; drug administration to patients with IBD, especially antibiotics that destabilize intestinal microbial communities, thus permitting *C. difficile* colonization and adherence; and nutritional status deterioration, which promotes *C. difficile* infection.

Furthermore, it has been suggested that the association between *C. difficile* and IBD may be mediated by a number of factors, including recurrent hospital admissions, a known risk factor for acquisition of *C. difficile* and CDI; drug administration to IBD patients, especially antibiotics that destabilize the intestinal microbial communities, thus permitting *C. difficile* colonization and adherence; immune system and nutritional status alteration; and genetic predisposition [19].

Following its colonization in the intestine of IBD patients, *C. difficile* secretes two types of potent exotoxins: toxin A (“enterotoxin”) and toxin B (“cytotoxin”), which bind to receptors on intestinal epithelial cells. These secretions activate a cascade of events and trigger the expression of pro-inflammatory cytokines and leukotrienes, inducing apoptosis of gut epithelial cells and increasing the permeability of the intestinal mucosa, which, in turn, can have a role in the pathogenesis of IBD [48].

For the treatment of IBD, a number of studies have investigated the applicability of traditional oriental medicines as an alternative to commonly used Western drugs such as anti-bacterial, anti-inflammatory, and immunosuppressive agents [49], which can produce a lot of side effects in patients. PWS, a herbal formulation, is reported to be an effective medicine for treating common gastrointestinal disorders [43,50]. However, the effectiveness of PWS treatment on IBD patients, especially those with *C. difficile* infection, has not been previously evaluated. Thus, in the present study, we investigated the effects of PWS in a mouse model of IBD. Our results revealed that PWS treatment can exert a potentially beneficial effect on DSS + CD-induced colitis, as reflected by the observed changes in clinical symptoms and pathophysiology such as improvement in stool-types disease activity index (DAI) scores and amelioration of shortening of colonic length. These results are consistent with the outcomes reported in previous clinical studies in which PWS treatment produced a significant improvement in clinical symptoms in up to 93.0% of patients suffering from ulcerative colitis [51]. Our study also demonstrated increases in both the size and weight of spleen in mice in response to DSS or DSS + CD treatment. This is in agreement with earlier investigations reporting a close relationship between spleen size and severity of DSS-induced colitis [52,53]. Additionally, our results showed a greater size and higher weight of spleen in the DSS + CD group compared with the DSS group. Although the differences were statistically insignificant, it can be surmised that spleen damage in the DSS + CD group was more serious than that in the DSS group. However, both the size and weight of the spleen in DSS + CD + PWS group were markedly reduced compared with that of the DSS + CD group, further indicating a beneficial effect of PWS against colitis-induced complications.

It has been suggested that pro-inflammatory cytokines have a vital role in the course of colitis in both mice and humans [54]. An accumulation of evidence indicates that both the innate and adaptive immune responses participate in the initiation of an inflammatory condition and activation of diseases that are associated with an increase in interleukin IL-1β and a decrease in IL-10 and TGF-β levels [55,56]. Indeed, upregulation of IL-1 was observed in both ulcerative colitis and Crohn’s disease [57,58]. Furthermore, it has been revealed that in the inflamed mucosa of IBD, IL-1β, a key mediator of colonic inflammation initiation, has a crucial role in the pathogenesis of colitis [14]. Meanwhile, it has been reported that TGF-β and IL-10, which are widely expressed in mucous membranes and the lamina propria, exert anti-inflammatory and immune suppressive actions in the colons of IBD mice [56]. As expected, in our study, the expression of IL-1β was higher in both the DSS and DSS + CD groups than in the normal group. Additionally, our results are in agreement with previous reports showing decreased expressions of TGF-β and IL-10 owing to DSS treatment or IBD [59,60]. However, treatment of DSS + CD-induced colitis mice with PWS produces a reduction in the level of IL-1β and an increase in the expression of both IL-10 and TGF-β in the colonic tissue. This is in keeping with the potent anti-inflammatory activities of PWS [61], including its inhibitory effects on LPS-induced production of pro-inflammatory cytokines in lipopolysaccharide-treated rats. Furthermore, pretreatment with PWS has prevented the inflammatory response in an HCl/ethanol-induced gastric mucosal injury rat model [62].

The epithelium in the gut has an important role in the pathogenesis of IBD. Intestinal permeability is mainly maintained by TJs, which regulate the functioning of the epithelial barrier [63]. Increased intestinal permeability in association with downregulation of TJ strand numbers is a salient feature of animal colitis models [64]. Claudin-1 and occludin are the two main proteins that form the core part of the TJ and regulate ion selectivity and paracellular permeability [65]. In our study, colonic expressions of both occludin and claudin-1 were reduced in response to DSS or DSS + CD treatments. However, co-treatment of DSS + CD-treated mice with PSW caused a marked increase in the tissue levels of both of these TJ proteins. On the basis of that observation, it is conceivable that PWS can improve paracellular permeability as well as epithelial barrier function in DSS + CD-induced colitis by upregulating occludin and claudin-1 expressions.

The integrity of the intestinal barrier also depends on the status of the intestinal immune system, which is largely influenced by gut microbial homeostasis. A healthy gut microbial community has a vital role in the protection of the host against the invasion and systemic dissemination of pathogenic microorganisms [66]. An imbalance in gut microbiota homeostasis owing to changes in microbe composition can contribute to a number of intestinal diseases [67] including IBD [66]. In support of that observation, several lines of evidence have indicated that DSS-induced colitis is associated with a structural alteration in microbe population, as well as a decreased gut microbe diversity [68], which in turn can provide a favorable environment for *C. difficile* colonization [69].

In our study, the community structure of the gut microbiota was analyzed using T-RFLP, revealing that, at the phylum level, the population of *Bacteroidetes* was lower in the DSS + CD group than in both the DSS and normal groups. Although the differences were insignificant, a previous study showed that phylum distribution in IBD patients harbored significantly fewer *Bacteroidetes* than that in healthy subjects [70]. Furthermore, an inverse association has been reported between infection with *C. difficile* and abundance of *Bacteroidetes* [71]. In addition, we observed that the abundance ratio of the *Firmicutes/Bacteroidetes* phyla was higher in the DSS + CD group than in the normal group, supporting the findings of a previous study [72]. However, a significant decline in this ratio in the DSS + CD-treated mice was seen upon co-treatment with PWS.

At the species level, we observed an enrichment in *E. coli* abundance in response to treatment with DSS or DSS + CD, although the chance was significant only in the latter treatment. Several reports have revealed that the relative abundance of *E. coli* increases as a result of inflammation of the intestinal tract [73,74,75,76]. Furthermore, DSS treatment was shown to promote colonization with Shiga toxin-producing *E. coli* [77]. Notably, in our study, the relative abundance of *E. coli* was observed to be lower in the DSS + CD + PWS group than in both the DSS and DSS + CD groups. In the present study, *Clostridium* cluster IV species showed a lower relative abundance in all treatment groups compared with the normal group, in support of the results in an earlier report [72]. A shifting in the abundance of *A. muciniphila* (S), a mucin-degrading bacterium living in the mucus layer of the intestinal tract, has been documented in IBD patients [67,78]. Our results revealed an increase in the abundance of *A. muciniphila* (S) in mice treated with DSS, in agreement with the results of a previous study [68]. However, treatment of DSS + CD-treated mice with PWS led to a decline in the abundance of *A. muciniphila*. In one of our previous studies, *Rhizoma Atractylodis Macrocephalae*, an active constituent of PWS, was shown to have a beneficial effect on the relative abundance of gut microbiota in rats treated with lipopolysaccharide [41]. In agreement with this finding, a polysaccharide from the rhizome of *Atractylodes macrocephala* was observed to significantly improve a disordered intestinal flora in rats; on that basis, it was surmised that this compound may be useful as an oral adjuvant in the regulation of intestinal flora [79]. A previous report also revealed that, in mice, supplementation with *Z. officinale*, another constituent of PWS, can modulate the composition of the gut microbiota, resulting in beneficial effects against a number of diseases including inflammation [80]. In agreement with above findings, the gross distributional shifts in the mouse gut microbial communities produced by DSS or DSS + CD treatment in our study were markedly counteracted by PWS treatment, suggesting that this herbal formulation has a protective effect against a disordered gut flora.

## 4. Materials and Methods

### 4.1. Animals

Seven-week-old male C57BL/6 mice (ethics project identification code: 2017-06165) were purchased from Daehan Biolink Co. Ltd. (Daehan Biolink, Eumseong, Chungbuk, South Korea). The animals were kept in a 12 h light/dark cycle under a constant temperature of 20 ± 3 °C with free access to autoclaved distilled water and standard chow. The animals were maintained under these conditions for one week to become acclimated. All procedures for animal experiments were performed in accordance with the principles outlined in the “Guide for the Care and Use of Laboratory Animals” (Institute for Laboratory Animal Research, Committee for the Update of the Guide for the Care and Use of Laboratory Animals, National Research Council of The National Academies, USA; The National Academies Press: Washington, D.C., 2011) and approved by the “Institutional Animal Care and Use Committee” of Dongguk University.

### 4.2. Preparation of C. difficile

*C. difficile* (KCTC 5009) was purchased from the Korean Collection for Type Cultures (Korean Collection, Daejeon, South Korea). Culturing and strain typing of this bacterial strain were performed as previously reported [64]. Briefly, *C. difficile* was incubated in a taurocholate–cefoxitin–cycloserine–fructose agar (TCCFA) plate for 48 h at 37 °C in an anaerobic chamber (Thermo Electron Corporation, Beverly, MA, USA). Eight single colonies from each plate were picked using sterile inoculating loops that were struck in quadrants on agar plates containing brain heart infusion medium supplemented with yeast extract (BHIS: 37 g brain heart infusion, 5 g yeast extract, 0.1% (*w*/*v*) l-cysteine, 15 g agar per liter). Cultures were grown under anaerobic conditions at 37 °C for 5–7 days. Sporulation was induced on media containing BHIS and SMC (90 g Bacto Peptone, 5 g protease peptone, 1 g NH_4_SO_4_, 1.5 g Tris base, and 15 g agar per liter), at 70% SMC and 30% BHIS (70:30 media, 63 g Bacto Peptone, 3.5 g Protease Peptone, 11.1 g BHI, 1.5 g yeast extract, 1.06 g Tris base, 0.7 g NH_4_SO_4_, 15 g agar per liter) [81]. Spores were harvested by centrifugation for 15 min at 3000× *g* and were washed with ice-cold PBS five times after isolation. The spores were then incubated at 70 °C for 20 min to ensure that no residual vegetative cells survived. Viable spores were counted by plating as colony forming units (CFU)/mL on TCCFA to determine the challenge dose [82].

### 4.3. Preparation of PWS

PWS is a formulation of six herbal medicines, which is prepared by mixing ground forms of *Atractylodes lancea*, bark of *Magnolia officinalis*, *Citrus reticulata* Blanco, *Glycyrrhiza uralensis, Zingiber officinalis*, and *Ziziphus jujube* Mill. at a ratio of 29:14:20:9:14:14 (weight, Table 1). All the crude-dried herbs used to make PWS were purchased from the Dongguk University Ilsan Medical Center (Goyang-si, Gyeonggi-do, South Korea). Initially, the herbal mixture was pulverized into a coarse powder, which was allowed to steep in 30% ethanol for 15 min. The mixture was then refluxed twice in 300 mL of 30% ethanol for 1 h. Two batches of filtrates were mixed together and, from this preparation, the solvent was completely removed by applying vacuum at 40 °C. The residue was then subjected to freeze-drying (IlshinBioBase, Dongducheon, Gyeonggi, South Korea) to yield the final PWS powder product, which was stored at −20 °C until use. The herbal powder was dissolved in PBS (pH 7.4) prior to dosing of experimental animals via oral administration.

### 4.4. Mice Model of Colitis and Treatment Regimen 

Forty-nine male mice (eight weeks of age, body weight: 20–23 g) were randomly assigned to one of four groups: (1) Normal (*n* = 12); (2) DSS (*n* = 12); (3) DSS + CD (*n* = 12); (4) DSS + CD + PWS (*n* = 13).

Prior to treatment, all mice were provided with free access to chow and distilled water as drinking water for two days (days 1 and 2; Figure 1A). Then, with the exception of the normal group, all mice received 2.5% (*w*/*v*) DSS (MP #161110, MW = 35–50 kDa, ICN Biomedicals Inc. Aoraro, OH, USA) in drinking water for seven days (day 0 to day 6) in order to produce DSS-induced acute colitis in the animals. In the DSS + CD group, the DSS-fed animals were orally administered with 10^7^ CFU of *C. difficile* spores twice over the period of treatment (days 0 and 3). In the DSS + CD + PWS group, the DSS + CD-treated mice received PWS (669.1 mg/kg/day) via oral gavage until the end of the experimental period (days 2 to day 7). During that same period, the mice in the normal, DSS, and DSS + CD groups were orally administered with the same volume of PBS without PWS.

### 4.5. Tissue Sampling, Histology, and Measurement of Disease Activity

Evaluation of the DAI in the mice was performed by measuring the clinical progression of the disease. An investigator blinded to the well-established DAI scoring system was assigned to this study. Animals underwent daily examination for evaluation of health status, especially diarrhea, and other symptoms. Bodyweight of the animals was measured every other day (days −2, 0, 2, 4, and 6; Figure 1A) and stool samples were collected four times prior to the termination of the study (days 0, 2, 4, and 6). The assigned DAI score was dependent upon the characteristics of the stool and was based on the combination of two types of stool indicators: stool consistency and fecal bleeding (Table 2). The DAI scores ranged from 0 (normal) to 3 (maximal disease activity).

Following termination of the experimental schedule on day 7, mice were sacrificed under anesthesia (Zoletil 50, Virbac Animal Health, Carros, France). The entire colon (from cecum to anus) and the spleen were removed and their lengths were measured. The tissues were then washed with PBS (pH 7.4), dried, and weighed. The cecum was then excised and the colon length and weight were determined. The colon was cut into slices and some of them were immediately submerged in RNAlater™ Stabilization Solution (Invitrogen, Carlsbad, CA, USA) in sterile tubes followed by snap freezing in liquid nitrogen and storage at −80 °C for future RNA analysis. Some of the other colonic fragments were also rapidly frozen in liquid nitrogen and stored at −80 °C for future protein analysis by Western blotting. The remaining colonic tissue slices were fixed in 4% paraformaldehyde overnight and embedded in paraffin blocks for histological processing. In order to proceed for hematoxylin and eosin (H&E) staining, the tissues were sectioned to 5 μm thickness using a microtome (Leica, Nussloch, Germany) and placed on positively charged glass slides (Leica Biosystem, Richmond, IL, USA), followed by deparaffinization with xylene (Duksan, Ansan, Gyunggido, South Korea) and dehydration through an increasing ethanol concentration gradient. The tissue sections were stained with hematoxylin solution (EMD Millipore, Darmstadt, Germany) and eosinY solution (0.5%) alcoholic (Biosciences, St. Louis, MO, USA) and then mounted on cover slips. The stained tissues were examined under an inverted light microscope (Olympus, Tokyo, Japan) at 100×, 200×, and 400× magnifications. Microscopic images were captured using a digital camera (Olympus, Tokyo, Japan). For Alcian blue (AB)-staining [83], the blocked tissues were sectioned to 5 μm thickness and dehydrated as stated above. The sections were placed on positively charged glass slides, dried, deparaffinized with xylene, and rehydrated in a decreasing ethanol series. The tissue sections were then stained with AB reagent (Alcian blue, 8GX, Sigma Aldrich, St. Louis, MO, USA) and examined under the inverted light microscope at 200× and 400× magnifications. The mucus thickness, AB-positive area, crypt depth, and the number of goblet cells were determined from the images using dedicated image analysis software (Image-Pro Plus 6.0, Media Cybernetics, Inc., Rockville, MD, USA). For the first two of the above-mentioned parameters, analyses were performed using 3–4 randomly selected fields per colonic section. From each animal, two sections were randomly chosen for these measurements. Crypt depth was measured from the base of the villus to the mucosa. Five crypts were randomly selected from different fields in each colonic section to determine the mean crypt depth of each animal. Goblet cells were counted in all crypts present in an entire colonic section; three sections per animal were randomly chosen for this measurement.

### 4.6. Extraction of Bacterial DNA from Feces

Bacterial DNA was extracted from fresh stool samples that were collected on the last day of the treatment schedule; extraction was performed using the QIAamp^®^ Fast DNA Stool Mini Kit (Qiagen, Hilden, Germany) per the kit manufacturer’s instructions. A previous study reported that DSS contamination of colonic tissue could inhibit the amplification of genomic DNA [84]. To avoid that complication, DNA was purified by precipitating with lithium chloride, a process based on the technique developed by Emilie et al. [48]. 

### 4.7. Purification of DNA by Lithium Chloride

The extracted DNA solution was incubated on ice for 2 h with a 0.1 volume of 8 M LiCl (Sigma Aldrich, Saint Louis, MO, USA) prepared in DNase/RNase-free water (Life Technologies, Carlsbad, CA, USA). The mixture was then centrifuged at 13,000 r/min for 30 min at 4 °C. The supernatant was discarded and the DNA pellet dissolved in 200 μL DNase/RNase-free water. That procedure was repeated once and the final obtained DNA pellet was incubated at −20 °C for 30 min, after which a 0.1 volume of 3 M sodium acetate (pH 5.2) and 2 volumes of absolute ethanol were added. The mixture was centrifuged at 13,000 r/min for 30 min at 4 °C. The supernatant was discarded and the DNA pellet was washed with 70% ethanol and centrifuged at 13,000 r/min for 30 min at 4 °C. The supernatant was discarded and the DNA pellet dried and dissolved in 10–50 μL of DNase/RNase-free water.

### 4.8. 16S rRNA-Based Terminal Fragment Length Polymorphism (T-RFLP) Analysis of Gut Microbial Community

The purified bacterial DNA from feces was amplified via polymerase chain reaction (PCR) using an AccuPower PCR PreMix kit (Bioneer, Seoul, South Korea) and broad-ranged primers: 27F-FAM (5′-AGAGTTTGATCCTGGCTCAG-3′) and 1492R (5′-GGTTACCTTGTTACGACTT-3′) (Macrogen, Seoul, South Korea). The conditions for PCR amplification reactions were as follows: initial denaturation for 3 min at 94 °C; 30 cycles of amplification encompassing denaturation at 94 °C for 1 min, annealing at 53 °C for 45 s, and elongation at 72 °C for 2 min followed by a final extension at 72 °C for 10 min. The PCR products were purified using a PCR purification kit (BIOFACT, Seoul, South Korea) according to the kit manufacturer’s protocol, and the purified products were subjected to digestion with restriction enzyme Hap II (Takara, Kyoto, Japan). Finally, the digested DNA products were submitted to Macrogen Korea (Macrogen, Seoul, South Korea) for T-RFLP-based analysis of the microbial population using a 3730XL DNA analyzer (BigDye v3.1, Macrogen, Seoul, South Korea). The T-RFLP electropherograms were visualized using GeneScan 3.7 software (Thermo Fisher Scientific, Carlsbad, CA, USA) and analyzed using XLStat software (Addinsoft, Brooklyn, NY, USA).

### 4.9. Analysis of Inflammatory and Anti-Inflammatory Markers and Tight Junction Proteins by Western Blotting

Stored colonic tissues were homogenized on ice in RIPA buffer (Biosesang, Gyeongsangnam-do, South Korea) containing cOmplete™, Mini, EDTA-free protease inhibitor (Roche Diagnostics GmbH, Mannheim, Germany), and the Xpert phosphatase inhibitor cocktail (GenDEPOT LLC, Houston, TX, USA). The homogenates were centrifuged at 13,000 r/min for 15 min at 4 °C, after which the supernatants were collected. The protein concentrations of the homogenates were measured using a Pierce^®^ BCA protein assay kit (Thermo Scientific, Rockford, IL, USA) according to the kit manufacturer’s instructions. Forty micrograms of proteins were resolved by SDS gel electrophoresis on 15% acrylamide–bisacrylamide gels using 80–100 V for 80 min and then transferred onto 0.45 μm polyvinylidene fluoride (PVDF) membranes (Amersham^TM^, GE Healthcare, Dornstadt, Germany) using a Mini Trans-Blot^®^ electrophoretic transfer cell device (BioRad, Hercules, CA, USA). The membranes were then blocked with Tris-buffered saline (TBS, Sigma Aldrich, Saint Louis, MO, United States) containing 0.1% Tween 20 (TBST) and 5% skim milk (Becton Dickinson, Sparks, MD, USA) for 1 h. Following this, the membranes were incubated overnight with the appropriate primary antibody at 4 °C as follows: anti-IL-10 (sc-365858, Santa Cruz, Baker, OR, USA), anti-TGF-β1 (sc-130348, Santa Cruz, Baker, OR, USA), anti-IL-1β (CSB-PA003023, CUSABIO, Wuhan, China), anti-occludin (sc-133256, Santa Cruz, Baker, OR, USA), or anti-claudin-1 (sc-166338, Santa Cruz, Baker, OR, USA). The immunoblots were then washed three times in TBST and incubated for 1 h with horseradish peroxidase (HRP)-conjugated anti-mouse or anti-rabbit Immunoglobulin G (IgG) secondary antibodies (Santa Cruz, Baker, OR, USA) depending on the origin of the primary antibody. The housekeeping protein β-actin was used as a loading control for the samples. The immunoreactive bands were traced on a Las3000 imaging system (Fujifilm, Tokyo, Japan) using Supex Enhanced chemiluminescence (ECL) reagent (Neuronex, Seoul, South Korea). Subsequently, the membranes were stripped in a buffer (62.5 mM Tris-HCl (pH 6.7) containing 2% SDS and 100 mM β-mercaptoethanol) and re-probed with anti-β-actin antibody (Santa Cruz, Baker, OR, USA) and processed identically as described above.

### 4.10. Statistical Analysis

All data are expressed as means ± standard error (S.D.). The *p*-values were determined using a two-tailed, unpaired Student’s *t*-test, or a repeated-measures analysis of variance (ANOVA) with Tukey’s correction for multiple comparisons.

## 5. Conclusions

Our study revealed that PWS treatment can ameliorate DSS + *C. difficile*-induced IBD in mice, as evidenced by the prevention of diarrhea, shortening of colon length, decrease in spleen size and weight, protection of TJ proteins, and improvement in the profiles of pro-inflammatory and anti-inflammatory cytokines. Such protective effects of PWS are likely to be, at least in part, mediated via restoration of balance in gut microbial communities.

## Figures and Tables

**Table 1:** Composition of *Pyungwi-san*.

Botanical Name	Pharmaceutical Name	Approximate Amount (%)
*Atyractylodes lancea*	rhizoma atractylodis	29
*Magnolia officinalis*	cortex magnoliae officinalis	14
*Citrus reticulata Blanco*	Pericarpium citri reticulata	20
*Glycyrrhiza uralensis*	radix glycyrrhizae uralensis	9
*Zingiber officinalis*	rhizoma zingiberis officinalis recens	14
*Ziziphus jujuba mill*	fructus zizyphi jujubae	14

**Table 2:** Criteria within the disease activity index (DAI) scoring system ^§^.

Score	Diarrheal Stool Score	Bloody Stool Score
0	Normal stool	Normal color
1	Mildly soft stool	Dark brown
2	very soft stool	Reddish
3	watery stool	Bloody

**^§^** The DAI scoring was based on a combination of two types of evaluation of stool: stool consistency and fecal bleeding. The DAI score ranges from 0 (normal) to 3 (maximal activity).
